# Optical Gain in Semiconducting Polymer Nano and Mesoparticles

**DOI:** 10.3390/molecules26041138

**Published:** 2021-02-20

**Authors:** Mark Geoghegan, Marta M. Mróz, Chiara Botta, Laurie Parrenin, Cyril Brochon, Eric Cloutet, Eleni Pavlopoulou, Georges Hadziioannou, Tersilla Virgili

**Affiliations:** 1 Department of Physics and Astronomy, University of Sheffield, Sheffield S3 7RH, UK; mark.geoghegan@newcastle.ac.uk; 2 IFN-CNR, Dipartimento di Fisica, Politecnico di Milano, 20132 Milan, Italy; marta.mroz@polimi.it; 3 SCITEC-CNR, Istituto di Scienze e Tecnologie Chimiche “Giulio Natta”, 20133 Milan, Italy; chiara.botta@scitec.cnr.it; 4 Laboratoire de Chimie des Polymères Organiques (LCPO) UMR 5629, CNRS-Université de Bordeaux-Bordeaux INP, CEDEX, 33607 Pessac, France; laurie.parrenin@gmail.com (L.P.); Cyril.Brochon@enscbp.fr (C.B.); Eric.cloutet@enscbp.fr (E.C.); epavlopoulou@iesl.forth.gr (E.P.); hadzii@enscbp.fr (G.H.)

**Keywords:** transient absorption spectroscopy, conjugated polymers, stimulated emission, nanoparticles, mesoparticles

## Abstract

The presence of excited-states and charge-separated species was identified through UV and visible laser pump and visible/near-infrared probe femtosecond transient absorption spectroscopy in spin coated films of poly[*N*-9″-heptadecanyl-2,7-carbazole-*alt*-5,5-(4,7-di-2-thienyl-2′,1′,3′-benzothiadiazole)] (PCDTBT) nanoparticles and mesoparticles. Optical gain in the mesoparticle films is observed after excitation at both 400 and 610 nm. In the mesoparticle film, charge generation after UV excitation appears after around 50 ps, but little is observed after visible pump excitation. In the nanoparticle film, as for a uniform film of the pure polymer, charge formation was efficiently induced by UV excitation pump, while excitation of the low energetic absorption states (at 610 nm) induces in the nanoparticle film a large optical gain region reducing the charge formation efficiency. It is proposed that the different intermolecular interactions and molecular order within the nanoparticles and mesoparticles are responsible for their markedly different photophysical behavior. These results therefore demonstrate the possibility of a hitherto unexplored route to stimulated emission in a conjugated polymer that has relatively undemanding film preparation requirements.

## 1. Introduction

Semiconducting polymer films may be a viable alternative to inorganic materials when high performance is less important than cost. Polymers have a significant advantage in that they may be solution processed, which permits film preparation routes such as spin coating, doctor blading, and drop casting. However, polymers need not be dissolved in solution but rather can be dispersed in a liquid medium as latex particles before being cast as films. This is appropriate for certain synthetic routes, such as emulsion polymerization or nanoprecipitation, which result in a particle suspension [1]. These are all compatible with various cross-coupling routes that can be used to synthesize conjugated polymers [2,3]. This wide choice of synthesis and film preparation routes allows fine tuning of the morphological and structural properties of the film. Semiconducting polymer particles can be optimized for light emission [4,5], energy generation [6,7], or phototherapies [8,9], among other purposes [10,11,12].

The optoelectronic properties of polymer films depend, at least indirectly, on their morphology as well as their electronic structure. Because optical and electronic properties are inextricably linked, electronic and excitonic transport need to be optimized for the required optical properties. For main-chain conjugated polymers, charge transport benefits from both interchain and intrachain phenomena and also from a good degree of crystallization. Optical properties, however, are less affected by crystallization than transport properties but perturbing the conjugation length of even rigid polymers is known to affect them [13]. Indeed, where comparisons between amorphous and crystalline polymers have been made, it is noted that changes in optical properties are due to the concomitant change in conjugation length as opposed to chain ordering [14,15].

Semiconducting polymer films that display stimulated emission often have excellent electroluminescent properties, and so effort spent optimizing optical gain is often performed with wider applications in mind [16]. Although optical gain in semiconducting polymer films has been known for some time [17] and is not rare, it is not commonplace either, and some thought is needed to achieve it. Interchain interactions are often a means of quenching excitations, so control of the photophysics of conjugated polymers can be obtained by enhancing single chain properties through dilution in a matrix of an amorphous polymer [18], encapsulation in nanochannels [19], or through the use of an appropriately structured film [20]. The separation of semiconducting polymers from each other (dilution) does not allow for good electronic transport, but other work [21] showed that, by mixing different polyfluorenes, it was possible to have films of semiconducting polymers with both optical gain and excellent charge transport properties. Further, alternative, approaches include mimicking the effects of dilution by adding a small amount of dimethyl phenylene into polyfluorene chains [22] or by the supramolecular separation of chains by threading cyclodextrin rings around the conjugated polymer to create polyrotaxanes [23].

Films are usually produced by spin coating or doctor blading from a polymer solution, which give rise to good uniformity and control of thickness. Rough films may result in weaker optical properties due to the scattering of light by the film surface. Such scattering may be exacerbated in particle films, particularly for particles of optical dimensions. Nevertheless, it could be reasonably argued that nanoparticle films convey some advantages because confinement inherent in the particles allows some element of dilution by enhancing intrachain properties due to the extended conjugation length with respect to interchain interactions. However, transient absorption spectroscopy revealed no evidence of stimulated emission in an aqueous nanoparticle suspension of poly[*N*-9″-heptadecanyl-2,7-carbazole-*alt*−5,5-(4,7-di-2-thienyl-2′,1′,3′-benzothiadiazole)] (PCDTBT) [24]. In contrast, a mesoparticle suspension of PCDTBT in propanol did show stimulated emission in the infrared region of the spectrum. Here, transient absorption is used to show that the photophysics of PCDTBT mesoparticle films (of diameter ~450 nm) is very different to those of nanoparticle films (~50 nm), and in particular, the mesoparticles exhibit good optical gain. Nanoparticles on the other hand exhibit photoinduced absorption due to the formation of charged states, rather than stimulated emission. Both polymers used for these films exhibit the same optical properties when spin cast from chloroform solution to form a continuous PCDTBT film. PCDTBT films are largely amorphous when spin coated from chlorobenzene solution [25], although there is some evidence of limited order when cast from chloroform [26]. These results show that it is possible to create good optical gain by the preparation route used to synthesize the films, rather than by using elaborate means to dilute semiconducting polymers.

## 2. Results

**Figure 1:**
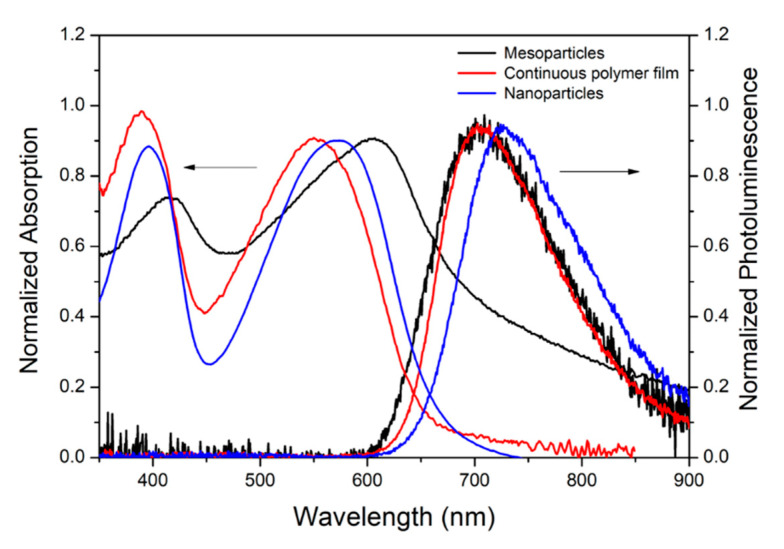
Absorption and emission (rightmost peaks) spectra of the continuous polymer (red line), mesoparticle (black line), and nanoparticle (blue line) spin coated films.

Figure 1 shows the absorption and emission spectra of the spin-coated nanoparticle, mesoparticle, and continuous polymer films taken as reference. The absorption spectrum of the continuous polymer film (red line) exhibits two broad transitions with peaks at 390 (3.19 eV) and at 550 nm (2.26 eV). In agreement with earlier work [24,27,28] the two bands are identified as the π–π*-transition of the first and second excited singlet states (S_1_ and S_2_). The emission spectrum shows a broad and unstructured emission between 620 (2 eV) and 920 nm (1.35 eV) (red line), peaking at 703 nm (1.77 eV). The spectra of the nanoparticles (blue lines) are very similar to that of the continuous polymer film, with a red shift of around 65 meV for both peaks in both absorption and emission. This is likely to indicate a more planar chain conformation and an increased conjugation length [29,30].

The absorption spectrum of the mesoparticle film (black line in Figure 1), prepared from a lower molar mass polymer, is quite different from those previously reported for PCDTBT [27,28]. In contrast to what would be expected [31], both transition peaks are red-shifted by around 150 meV, and also their relative intensity is changed, with the transition at 607 nm being more intense than that at 415 nm. The long wavelength absorption tail is likely to be due to the Rayleigh scattering of light from the 450 nm particles [32]. Despite the red-shifted absorption, the photoluminescence (PL) emission (black line in Figure 1) is similar to the one of the polymeric film with the main peak at 703 nm, resulting in an evident reduction of the Stokes shift.

**Figure 2:**
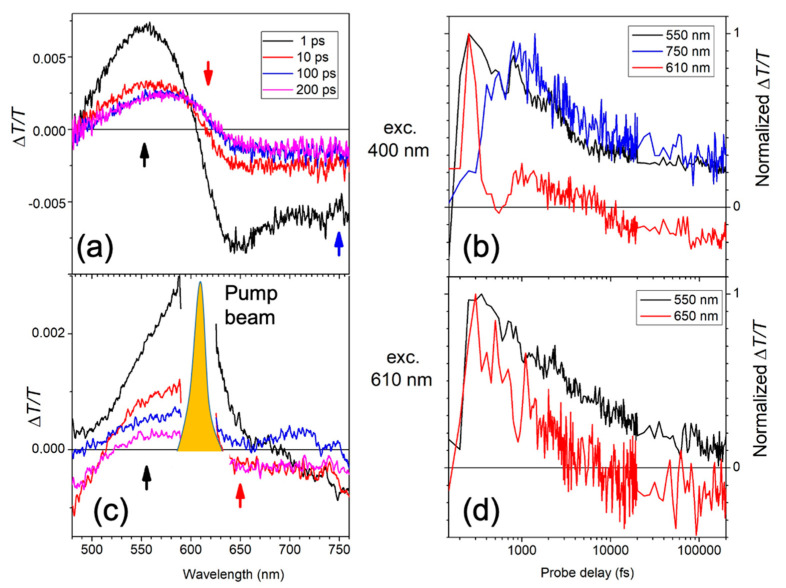
Left panel: Transient absorption spectra at different probe delays for the continuous polymer film at the different wavelengths of pump excitation: 400 nm (**a**) and 610 nm (**b**). The arrows correspond to different wavelengths (black, 550; red, 650; and blue 750 nm) at which the temporal decays are shown in (**b**,**d**). Right panel: Time decays at the two different pump excitation wavelengths: (**b**) 400 and (**d**) 610 nm. The shape of the 610 nm pump beam is also shown in (**c**).

The left panel of Figure 2 presents the pump-probe spectra at different probe delays for the continuous polymer film after excitation at 400 nm (Figure 2a) and at 610 nm (Figure 2c). After excitation at 400 nm the pump-probe spectrum shows the presence of an initial bleaching band centred around 550 nm (low energy peak in the absorption spectrum) and a photoinduced absorption band between 650 nm and 780 nm attributed to charged states [24]. In the first ~10 ps a red shift of the bleaching is present (Figure 2a, red line) indicating an energy migration towards the lower energetic sites of the absorption spectrum. In this spectral region an overlap between the positive bleaching signal and the negative one due to charge formation is also present. To highlight the charge formation, the dynamic*s* at 750 nm are considered (Figure 2b), which shows a formation time of around 1 ps.

The low energetic sites at 610 nm were also excited. The pump-probe spectrum shows an instantaneous bleaching of the high and low energetic excited states (see red and black line in Figure 2d) inducing an initial enlargement of the bleaching band of around 60 nm (0.19 eV). Nevertheless, even with this pump excitation the photoinduced absorption (PIA) band due to charged states is present with a low intensity. It is therefore possible to create charges even for excitation of the tail of the polymeric film of the absorption spectrum. In this way fewer charges are created, but a gain region in the continuous polymer film is nevertheless not observed.

**Figure 3:**
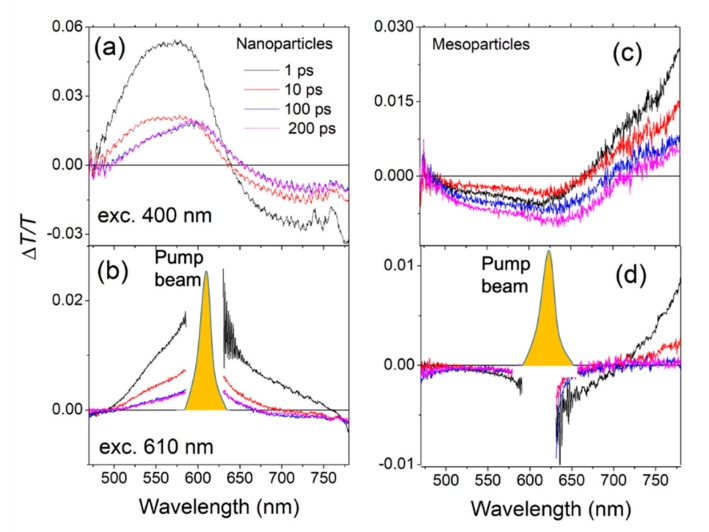
Transient absorption spectra at different probe delays for nanoparticle (**a**,**b**), and mesoparticle (**c**,**d**), films at two different wavelengths of pump excitation: 400 nm (**a**,**c**) and 610 nm (**b**,**d**). The ordinates for all axes are Δ*T*/*T* and the abscissae are wavelength.

The pump-probe spectra are presented at different probe delays for the nanoparticle film after excitation at 400 nm (Figure 3a) and at 610 nm (Figure 3b) and for the mesoparticle film (Figure 3c,d). The difference between the films is evident.

**Figure 4:**
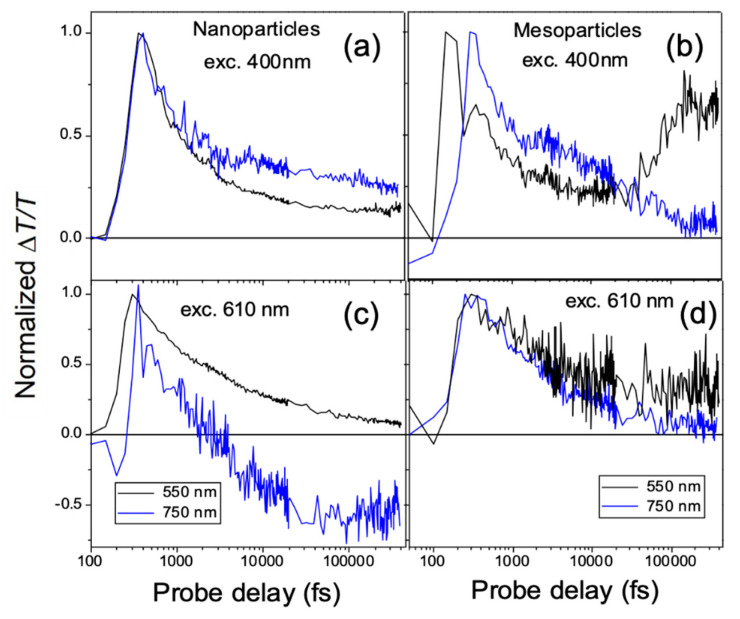
Time decays at different wavelengths for nanoparticle (**a**,**c**), and for mesoparticle (**b**,**d**), spin coated films at two different pump excitation wavelengths of 400 nm (**a**,**b**), and 610 nm (**c**,**d**) are displayed. The ordinates and abscissae for all axes are normalized Δ*T*/*T* and probe delay respectively.

The nanoparticle film is first considered. After excitation at 400 nm, the pump-probe spectrum shows the presence of an initial bleaching band centred at around 570 nm (low energy peak in the absorption spectrum) and a photoinduced absorption band at 700–800 nm attributed elsewhere to charged states [24]. The spectrum is similar to the one of the polymer film even if the dynamics show an instantaneous charge formation (blue line in Figure 4a) indicating a more efficient process. Even in this case there is a small red shift of the bleaching band toward low energetic states.

**Figure 5:**
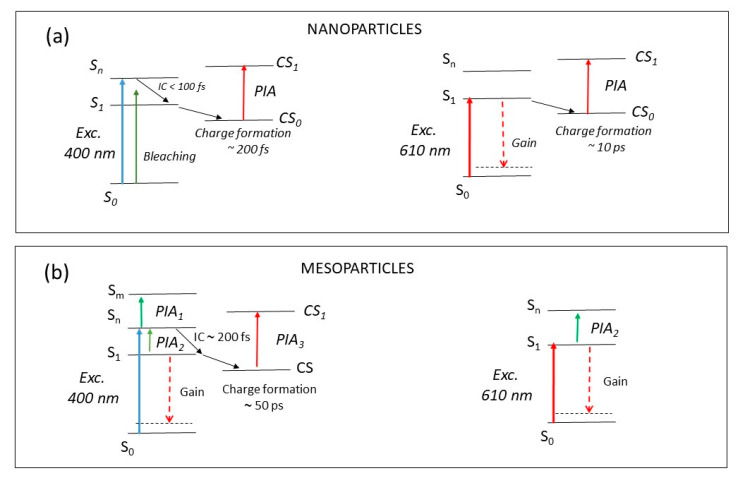
Schematic energy diagrams for the nanoparticle (**a**) and mesoparticle; (**b**) polymer films, where CS indicates the charged state and IC is an internal transition.

After excitation at 610 nm, a very different behavior was observed compared to that after 400 nm excitation. The positive band extended up to 750 nm indicating the presence of a long-lived gain region that is not present when exciting at 400 nm. It is therefore possible to have optical gain after excitation on the tail of the absorption spectrum. The direct excitation at 610 nm creates few charges (formation time ~10 ps, see Figure 4c), allowing the nanoparticles to have gain (see Figure 5a for the schematic energy diagram after each excitations).

Excitation of the mesoparticle films at 400 nm produces a large PIA band in the visible region and a positive band in the near-infrared region. As already seen in solution [24], the positive band is attributed to stimulated emission, while the instantaneous negative band present in the visible region was not present in the transient transmitted spectra of the solution. Looking at the decay traces (Figure 4b), the signal at 550 nm is instantaneously created. It has a rapid initial decay and then a recovery of the signal after ~200 fs. The positive signal at 750 nm is not instantaneously created and has a similar formation time of ~200 fs. The initial PIA band (*PIA*_1_) at 550 nm is attributed to the S*_n_*–S*_m_* singlet transition, it is followed by a fast internal conversion that increases the population of the state S_1_, which in turn produces the stimulated emission (gain) at 750 nm and a signal (*PIA*_2_) from S_1_ to S*_n_* (see the schematic energy diagram in Figure 5b) in the visible region. Moreover, after ~50 ps, a negative band centred at 650 nm (*PIA*_3_), attributed to the formation of charged states, overlaps with the initial photoinduced signal [24]. In the mesoparticle film, after excitation at 400 nm, this represents a different photophysics compared to the continuous and nanoparticle films: an internal transition from S*_n_* to S_1_, a strong gain signal in the near-infrared region, and slow charge formation are evident.

After excitation of the mesoparticle film at 610 nm, the pump-probe spectra are similar to the 400 nm excitation. A large negative band in the visible region and a positive one (gain) in the near infrared are present. The decay traces show that the signals at 750 and 550 nm are both instantaneously created and behave similarly (Figure 4d). This is then a direct excitation of the S_1_ state which produces a stimulated emission in the near infrared and *PIA*_2_ in the visible region (S_1_–S*_n_* transition). In this case, charges are not created. In the mesoparticle films, it is possible to have optical gain after excitation at both excitation wavelengths. After excitation at 400 nm, a 200 fs internal conversion brings the excitation to the lower energetic site from which stimulated emission is observed. The direct excitation at 610 nm allows an instantaneous emission with no charge formation.

## 3. Discussion

Before comparing the different films, it is important to recall that when these two particles are dissolved in a common solvent, and spin cast to form a continuous film, they exhibit the same photophysical properties [24]. This observation provides strong evidence that differences between the optical properties of the mesoparticle and nanoparticle films are due to size effects, rather than their differing molar masses.

The red shift in the absorption spectrum of the nanoparticle films, with respect to the continuous polymer films, can be associated to an increased conjugation length. The Stokes shift of ~0.49 eV (~150 nm), however, is similar for the two films, with the absorption and emission spectra red-shifted by the same amount. The mesoparticle films, on the other hand, display a much smaller (0.27 eV) Stokes shift, leaving the emission spectrum in the same position as that for the uniform film. The differences in the steady-state absorption-emission spectra of polymeric assemblies are commonly analyzed within an H/J-aggregate model [33,34]. As for the mesoparticle suspension [24] but also for the mesoparticle film absorption spectrum, the π–π*-transition of the first singlet state S_1_ is red-shifted with respect to the nanoparticle one while the PL peak is blue-shifted as expected for J-aggregate-like behavior, which is generally associated with stronger intrachain than interchain coupling [20,34]. The weak interchain interactions of the mesoparticle films are responsible for the limited charge formation observed here [27].

Stronger interchain interactions evident in the continuous and nanoparticle films explains the pump-probe spectra. The fast red shift in the bleaching, due to excitonic energy transfer [24], reveals the presence of molecular disorder within the continuous polymer and nanoparticle films. This red shift is not present in the mesoparticle films due to reduced exciton migration and/or greater molecular order in the mesoparticles.

Furthermore, charge formation in the mesoparticle films is suppressed at the longer (610 nm) excitation wavelength. Charge formation is very rapid for both the nanoparticle and continuous films. However, at the higher energy excitation, charge formation is observed in all films, although it is slowest in the mesoparticle film, where formation takes place on the order of 50 ps, compared with 1 ps (continuous film) and 200 fs (nanoparticle film). The reduced efficiency of charge formation in the mesoparticle films indicates of a more ordered system in these films, whereas the rapid charge formation in the other films (nanoparticle and continuous) supports the conclusion of a disordered molecular structure.

Optical gain is absent from the continuous polymer films at both excitation wavelengths, and also from the nanoparticle films at 400 nm. However, long-lived stimulated emission is observed following excitation at 610 nm for the nanoparticle films. This longer wavelength was not tested in the suspension. The mesoparticle films exhibit optical gain in the infrared at both wavelengths.

We cannot exclude optical gain existing in all films, but the nanoparticle and continuous polymer films all exhibit strong photoinduced absorption, which may overlap with the stimulated emission signal. Certainly, it has been possible to detect stimulated emission in the infrared in PCDTBT films spin-cast from chloroform, after excitation at 400 nm, and this signal strongly overlapped with that due to photobleaching [28].

It is unlikely that the particle size is the direct reason for the observed behavior, but rather the effect of the particle size on the chain conformation within the particles. This may also be a consequence of the synthetic route used to prepare the films. In the mesoparticle films, the chains are able to order in a way that gives rise to fewer interparticle interactions competing with intraparticle interactions and consequently less charge formation, which allows for an increased optical gain.

The phenomenological explanation of the optical properties of these films also applies to those of the nanoparticle and mesoparticle suspensions, where it was concluded that the mesoparticles have weaker interchain coupling and the optical properties, including stimulated emission, are likely to be due to a more J-aggregate-like behavior that is not observed for the nanoparticle suspension. Although the nanoparticle films revealed some stimulated emission when fewer charges were created after excitation at 610 nm, the longer wavelength excitation was not performed in the experiments on the suspensions, precluding a direct comparison.

## 4. Materials and Methods

Two routes to create PCDTBT particles were used. High molar mass nanoparticles were created as described previously [35]. PCDTBT (number average molar mass, *M*_n_ = 20.2 kDa with dispersity 2.2) was first synthesized by a Suzuki cross-coupling route [36] before being dissolved in chloroform. This was added to an aqueous solution of sodium dodecyl sulfate and sonicated to form a mini emulsion. The chloroform evaporated after heating at 70 °C, leaving an aqueous nanoparticle dispersion. The mean particle diameter was determined by transmission electron microscopy to be 50 nm.

PCDTBT mesoparticles were created as previously described [37]. PCDTBT (*M*_n_ = 4.5 kDa with dispersity 2.1) was synthesized by Suzuki cross-coupling polymerization in propanol solution with poly(vinyl pyrrolidone) added as a surfactant. By adjusting the quantity of surfactant, it was possible to adjust the particle size in the range 0.33–1.3 μm, with a virtually uniform size distribution. The films created in this work were made of 0.45 ± 0.05 µm particles.

For the continuous film, chloroform solutions (40 mg/mL) were prepared by dissolving the mesoparticles and then a film of the polymer was prepared by spin coating.

Film thickness was not a determining factor in these experiments but, in order to get a good optical signal during the transient absorption measurements, the mesoparticle films needed to be less than 1 µm thick. Nanoparticle and continuous films were substantially thinner, and they can be assumed to be of the order of ~100 nm that is typical for transient absorption measurements.

Absorption and emission spectra were acquired using a Shimadzu UV-3600 spectrophotometer (Shimadzu, Marne la-Vallée, France) and a Horiba Scientific Fluoromax-4 spectrofluorometer (Horiba, Palaissau, France), respectively. The excitation wavelength for the emission spectra was 390 nm.

Time-resolved measurements were performed using a homebuilt femtosecond pump–probe setup [24,38]. A Ti:sapphire regenerative amplifier (Libra, Coherent, CA, USA) was used as a laser source, delivering 100 fs pulses at a central wavelength of 800 nm with 4 mJ pulse energy at a repetition rate of 1 kHz. The second harmonic of the fundamental wavelength was used for the excitation at *λ* = 400 nm and for the excitation pulse at 610 nm, a single stage non-linear optical parametric amplifier, pumped at 400 nm was used. In order to minimize bimolecular effects, the excitation density was kept very low. White light generated with a 2 mm-thick sapphire plate was used as a probe in the visible-near infrared range from 450 to 780 nm. For spectrally resolved detection of the probe light, spectrographs and CCD arrays were used. The chirp in the white light pulse was taken into account during the analysis and evaluation of the two-dimensional (wavelength and time) Δ*T*(*λ*,*τ*)/*T* maps before extraction of the spectral and temporal data using homemade software. Overall, a temporal resolution of at least 150 fs was achieved for all excitation wavelengths.

## 5. Conclusions

Stimulated emission is a phenomenon which occurs in many conjugated polymers where photoluminescence is observed. However, more often than not, it competes with other processes such as charge creation or photobleaching and its detection and consequent utility is impeded. To this extent, researchers have gone to considerable lengths to create films which have limited interchain optical behavior. In this work, UV-visible absorption and photoluminescence spectroscopy, coupled with time-resolved transient absorption spectroscopy was used to demonstrate unambiguous optical gain in hard latex (450 nm) films of the conjugated polymer PCDTBT. Optical gain was weaker in nanoparticle films. It is concluded that conjugated polymer mini emulsions may be a viable route to creating systems with increased conjugation length. However, a full understanding of why such a preparation route increases the polymer conjugation length, or even whether it does or not, is lacking.

## Data Availability

Data are contained within the article.

## Sample Availability

Samples of the compounds are not available from the authors.
